# Targeted Synthesis of the Type-A Particle Substructure from Enzymatically Produced Eumelanin

**DOI:** 10.1021/acs.biomac.1c01390

**Published:** 2022-01-04

**Authors:** Anne Büngeler, Fabian Kollmann, Klaus Huber, Oliver I. Strube

**Affiliations:** † Institute for Chemical Engineering, University of Innsbruck, 6020 Innsbruck, Austria; ‡ Biobased and Bioinspired Materials, Paderborn University, 33098 Paderborn, Germany; § Department of Physical Chemistry, Paderborn University, 33098 Paderborn, Germany

## Abstract

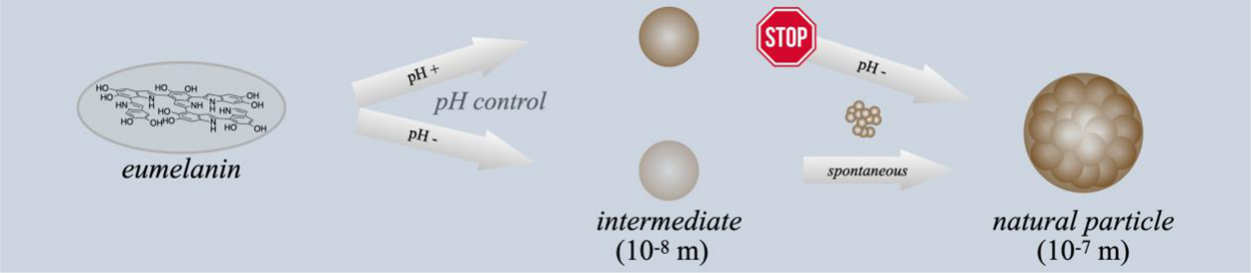

Eumelanin exhibits a defined supramolecular buildup that is deprived of at least three distinct particle species. To enable the full potential of its promising material properties, access to all particle types is crucial. In this work, the first protocol for the synthesis of the intermediate type-A particles in pure and stable dispersion form is described. It is found that aggregation of type-A particles into the larger type-B variant can be inhibited by a strict pH control during the synthesis. The exact influence of pH on the supramolecular buildup is investigated via a combination of time-resolved light scattering, electron microscopy, and UV–vis spectroscopy. It is observed that a rapid buildup of type-B particles occurs without pH control and is generally dominant at lower pH values. At pH values above 6.2 however, type-A particles are gained, and no further aggregation occurs. Even more, lowering the pH of such a stable type-A dispersion at a later stage lifts the inhibition and again leads to the formation of larger particle species. The results confirm that it is easily possible to halt the aggregation of eumelanin substructures and to access them in the form of a stable dispersion. Moreover, a profound additional understanding of the supramolecular buildup is gained by the in-depth investigation of the pH influence.

## Introduction

Melanins are a class of biological pigments with unique properties. The most important types are the black-brown eumelanin, reddish yellow pheomelanin, and neuromelanin. In addition to these natural representatives, a number of synthetic melanins and melanin-like structures are also known.^1,2^ The main natural task of the pigments is to protect against harmful UV radiation.^3,4^

Melanins are of high medical relevance, especially in melanoma research,^5,6^ but are also of outstanding interest in the field of materials science. Particularly, eumelanin inherits a large number of relevant material properties. The most important of these are radiation protection,^7,8^ radical scavenging properties,^9^ paramagnetism,^10,11^ targeted drug release,^12−14^ and hybrid electrical conductivity.^15,16^

This plethora of functionalities is unrivaled for any kind of biological material and derives from eumelanin’s distinct, molecular, and supramolecular structure. At the molecular level, enzymatically produced eumelanin pigments consist of chemically disordered oligomers with a variable monomer composition mainly made of the indolic derivatives 5,6-dihydroxyindole (DHI) and 5,6-dihydroxyindole-2-carboxylicacid (DHICA).^17^ The monomer synthesis and potentially also the polymerization are catalyzed by tyrosinase and other related enzyme variants.^3,18^ As the molecular structure of the various oligomers and the influence of the varying compositions of DHI and DHICA on the material properties are quite well understood, comparatively little is known about the supramolecular aspects.

**Figure 1:**
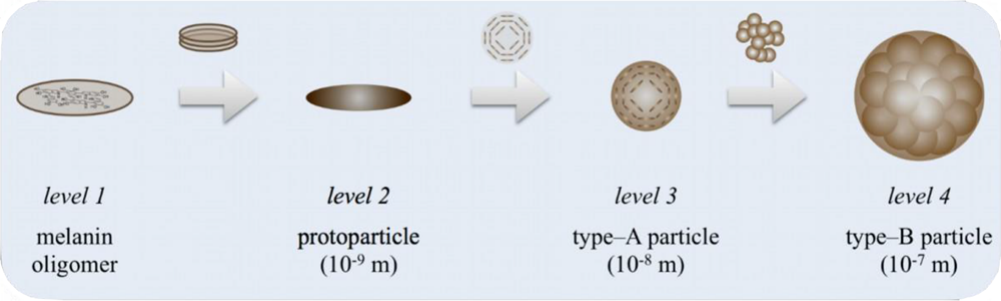
Schematic overview regarding the current state of knowledge on the supramolecular buildup of biomimetic eumelanin.

Both natural and biomimetic eumelanins are usually found in the form of spherical particles with a diameter of around 200 nm denoted as type-B particles.^19−23^ It was first proposed in the mid-1990s that these final type-B particles are built up according to a hierarchical aggregation mechanism.^16^ This has been further substantiated and updated in recent years. Currently, a four-level process with three different particle species is state of the art (Figure 1).^21,24^

In this model, the interaction between the individual oligomers represents the beginning of the supramolecular buildup that first leads to protoparticles, whose exact structure has not yet been clarified.^25,26^ In the second step, the protoparticles form the intermediate type-A particle, which might be arranged in an onion-like structure, although this information is based on one single original reference.^27^ Type-A particles finally aggregate further into the final type-B structure. The nature of this final aggregation step has previously been investigated in great detail,^21^ but many aspects regarding the supramolecular buildup have not been adequately clarified. However, understanding the buildup process of the melanin particles is crucial to fully harvest its potential for material applications. Specifically, producing the intermediate species in isolated form with the molecular buildup is one of the important quests in melanin research.^28^

Research on the supramolecular aspects recently gained new attraction with the introduction of the methodology of enzyme-mediated addressing. This technique enabled the first isolation of both intermediate particle types (type-A and protoparticle) by variation of enzyme mobility. However, due to the inherent goal of this approach, which is coating of the solid supports, the particles are not generated in dispersed form, which is limiting the applicability.^29,30^ Nevertheless, it was proven that it is possible to isolate the different particle species of melanin. Now this revelation has to be transferred to a process in a solution to gain access to stable particle dispersions. This work aims exactly for such a synthesis of stable dispersions of non-aggregated type-A particles made from biomimetic eumelanin.

To this end, we looked at the various reaction parameters of the biomimetic (enzyme-mediated) eumelanin synthesis. One parameter, whose influence stands out, is the pH value. As will be described in the following, the influence of pH on the supramolecular buildup is crucial. Still, this has never been investigated before. In the literature, the pH value has only been assigned an inferior role, so that very little is known or investigated on its influence on the melanin particle structure. There are investigations on the influence of pH on the properties of melanin such as paramagnetism and conductivity.^31^ Furthermore, natural and biomimetic eumelanin was redispersed, and the pH of the dispersions was adjusted using NaOH or HCl. The subsequent change in the pH of the final melanin particle dispersion however showed no influence on the existing particle structure.^16,31^

In contrast to this, we control the pH during the synthesis of melanin and can thus show its influence on the particle buildup mechanism. The aim of this work is to show that the pH value is a decisive and easily controllable parameter in the supramolecular buildup process to gain stable type-A particle dispersions.

## Experimental Section

### Materials

l-Dopa, tyrosinase from mushrooms (lyophilized powder, ≥1000 unit/mg solid), and MES buffer were purchased from Sigma-Aldrich. All experiments were carried out in ultrapure water (HPLC grade, specific conductivity ≤1 μS/cm) by VWR. All other chemicals were used as supplied, without further purification.

### Sample Preparation

#### Reference Experiment

First, an enzyme solution and an l-dopa solution were prepared in ultrapure water with concentrations of 0.1 g/L (850 units/L) and 0.2 g/L, respectively. After complete dissolution, 5 mL of the l-dopa solution was placed in a glass vial. Successively, 5 mL of the enzyme solution was put into the same glass vial to start the melanin reaction. After 10 s of gentle shaking, 2 mL of the sample was filtered with a PVDF filter (Millipore Millex-HV with a pore diameter of 0.45 μm) into the light scattering cuvette, and the measurement was started immediately. The final concentrations of the enzyme and l-dopa of all experiments amount to 0.05 g/L (425 units/L) and 0.1 g/L, respectively.

#### NaOH Experiments

The aqueous solutions of the two components were prepared analogously to the reference experiment. l-Dopa solution (5 mL) was placed in a glass vial, and then 5 mL of the enzyme solution was added to start the reaction. After 30 s during which the reaction mixture was gently shaken, the first amount of NaOH (0.1 M) was added. The second amount of NaOH was added 30 s later, and the sample was briefly shaken for a few seconds (Table 1). Finally, 2 mL of the sample was filtered with a PVDF filter into the light scattering cuvette, and the measurement was started immediately.

**Table 1:** Used Amounts of NaOH (0.1 M) during the NaOH Experiment Series

NaOH experiment	first amount of NaOH	second amount of NaOH	total amount of added NaOH
1. black curve	15 μL	20 μL	35 μL
2. blue curve	20 μL	20 μL	40 μL
3. red curve	20 μL	25 μL	45 μL

#### MES Experiments

The corresponding buffer solutions with MES were initially prepared as solvents. A 30 mM MES buffer with a pH of 4.8 was used to check the compatibility of the buffer with the melanin system, and a 15 mM MES buffer with a pH of 6.2 was prepared for type-A particle synthesis. The enzyme solution and the l-dopa solution were prepared in the corresponding MES buffer in the same way with the same concentrations as in the reference measurement described above. After complete dissolution of the two solutions, again, 5 mL of each solution was combined to start the reaction. For the light scattering measurement, the reaction solution was again filtered with a PVDF filter into a cuvette and the measurement was started immediately.

#### Reactivation of a Stable Type-A Dispersion Experiment

For the aggregation experiment from a stable type-A dispersion into the final form of type-B particles by lowering the pH from 6.2 to 4.8, a type-A dispersion was produced in 15 mM MES with pH 6.2 as described above. Melanin reaction solution (6 mL) was filtered into a light scattering cuvette with a PVDF filter immediately after the combination of the two starting solutions and left there for 24 h. Successively, 10 measurements of the aged solution were carried out to check the stability of the dispersion and the particle size.

### Light Scattering Setup

For all samples, 2 mL of the corresponding melanin reaction solution was added to the light scattering cuvette using a syringe with a PVDF filter (Millipore Millex-HV) with a pore diameter of 0.45 μm, and the measurement was started immediately. The time was monitored from the moment where the two components were combined, thereby defining *t* = 0. Light scattering experiments were done with the multi-detection laser light scattering system *ALV/CGS-3/MD-8.* A He–Ne laser with a wavelength of 632.8 nm was used as a light source. The system provides eight detectors that are positioned in an angular increment of 8°. This allows simultaneous and time-resolved dynamic and static light scattering. All samples were measured at a temperature of 25 °C. Typically, 10 s was applied to record an angular dependent, combined SLS and DLS measurement.

### Data Evaluation

To obtain the weight-averaged molar mass *M*_w_ and the *z*-averaged-squared radius of gyration *R*_g_^2^, the angular dependency of the scattering intensity, expressed in terms of the Rayleigh ratio Δ*R*_θ_, was evaluated with the following series expansion according to Berry:^32^
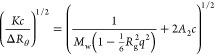
1*K* = (4π^2^*n*_solv_^2^/λ_0_^4^)(d*n*/d*c*)^2^ is the contrast factor with the wavelength of the laser λ_0_ = 632.8 nm in a vacuum, *n*_solv_ = 1.333 is the refractive index of the solvent, and (d*n*/d*c*) is the refractive index increment of the solute in solution*.* Due to the lack of a d*n*/d*c* value for melanin, a default value of d*n*/d*c* = 0.1 mL/g was applied. The quantity *c* is the mass concentration of l-dopa, *A*_2_ is the second virial coefficient, and *q* is the scattering vector, given by
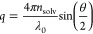
2with the scattering angle θ. A plot of  versus *q*^2^ gives  as the *y*-intercept and  as the slope of the linear regression. The factor 2*A*_2_*c* in eq 1 had to be neglected because an extrapolation of the scattering data to *c* = 0 was not possible in the aggregating and reacting melanin samples. Neglect of 2*A*_2_*c* is justified in light of the very small concentration of *c* = 0.1 g/L applied in the present work.

The dynamic light scattering experiments give the normalized field–time correlation function *g*_1_(τ), which was evaluated with a cumulant analysis:^33^
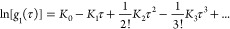
3*K*_0_ represents the signal-to-noise ratio, and *K*_1_ is the *z* average of the characteristic decay time, which is connected to the diffusion coefficient *D*_app_ via
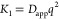
4The coefficient *K*_2_ is the variance of *K*_1_ and serves as an indicator for the polydispersity of the sample. *K*_3_ describes the asymmetry of the variance. The diffusion coefficients *D*_app_ were extrapolated to *q* → 0, according to eq 5
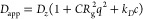
5The constants *C* and *k_D_* are the proportionality factors describing the angular and concentration dependency of *D*_app_, respectively. A concentration dependency of *D*_app_ had to be neglected because an extrapolation of the scattering data to *c* = 0 was not possible in a reacting and aggregating sample. The low concentration of *c* = 0.1 g/L in all experiments justifies the neglect of *k_D_c*. A plot of *D*_app_ versus *q*^2^ gives the *z* averaged diffusion coefficient *D_z_*, which can be inserted into the Stokes–Einstein equation^34^ to obtain the hydrodynamic radius. It represents the radius of a hydrodynamically equivalent sphere with the same diffusion coefficient as the particles under consideration:
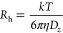
6In eq 6, *T* is the temperature, *k* represents the Boltzmann constant, and η is the viscosity of the solvent. Additional information about the structure of the particles can be obtained with the structure sensitive ratio ρ, which is defined by the geometric size parameter *R*_g_ and the hydrodynamically effective size *R*_h_.
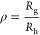
7The typical values for ρ are 0.77 in compact spheres and 1.55 for monodisperse linear chains.^35,36^

In addition to the cumulant analysis, the electric field–time correlation functions from DLS were interpreted by means of CONTIN analysis according to Provencher.^37^ The resulting size distributions of the melanin particles formed are displayed as an intensity-weighted distribution of the hydrodynamic radii.

### Scanning Electron Microscopy (SEM)

For SEM measurements analogous to the light scattering experiments, 2 mL of sample was taken and admixed with 1 mL of HCl (0.1 M) to terminate the reaction. After repeated washing with ultrapure water and carefully centrifuging, 0.2 mL of the samples was dried on a silicon wafer for subsequent investigation by SEM. The obtained samples were examined by means of electron microscopy using a ZEISS “Neon 40” scanning electron microscope. The pictures of the samples were obtained by applying the SE2-detector or the InLens detector at an acceleration voltage of 2 kV.

### UV/Vis Spectroscopy

The same procedure as for the light scattering was carried out with all UV/vis samples so that 2 mL of the melanin reaction solution was also filtered into a UV/vis cuvette, and the measurement was started immediately. UV/vis spectroscopy was performed with a THERMO SCIENTIFIC “Evolution 600” spectrometer using the time-resolved transmission method for the two fixed wavelengths. The transmission of the samples was measured in 30 s steps (analogous to the light scattering measurement) for the two different wavelengths of 475 and 633 nm.

### Time-Resolved pH Measurement

For the time-resolved pH measurements analogous to the light scattering experiments, identical samples were prepared as described above. In each case, 5 mL of the l-dopa solution was mixed with 5 mL of the enzyme solution to start the reaction. The samples were then mixed for about 30 s, and then the first amount of NaOH (0.1 M) was added. After a further 30 s, the second amount of NaOH was added (exact amounts of the added NaOH are shown in Table 1 above), the sample was briefly mixed by gently shaking it and then left to stand for the entire duration of the measurement without stirring. All the samples were measured in 10 s steps on a pH/mV conductivity meter from METTLER TOLEDO “SevenExcellence”.

## Results and Discussion

### Behavior of pH in the Reference Reaction

**Figure 2:**
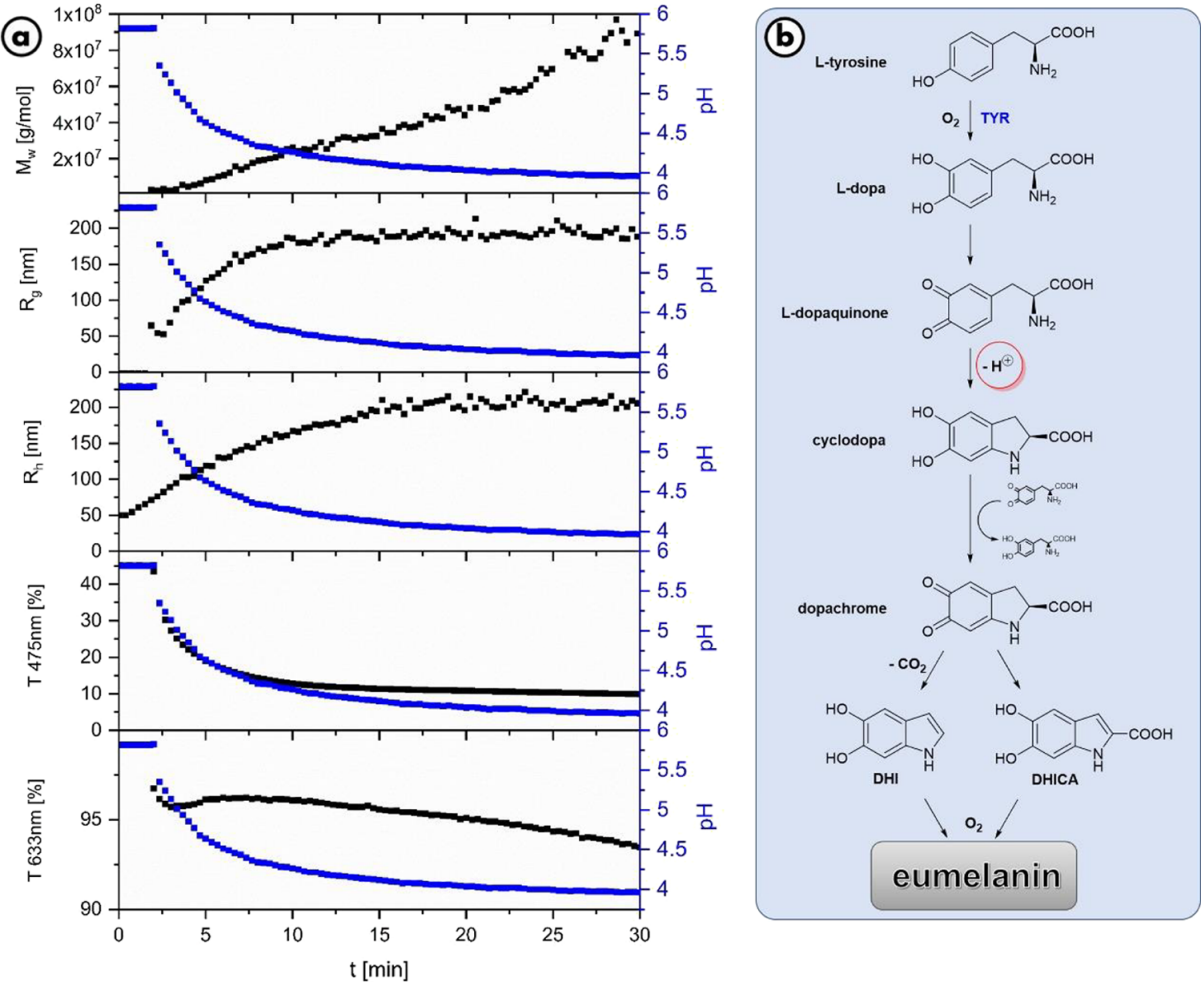
a.) Evolution of molar mass, radius of gyration, and hydrodynamic radius over time (black curves) during a reference experiment measured with time-resolved combined static and dynamic light scattering in comparison to the pH (blue curve), transmission at 475 nm, and transmission at 633 nm. Each experiment was performed with 0.1 g/L l-dopa and 0.05 g/L tyrosinase. b.) Mechanism for the molecular buildup of eumelanin precursors based on literature.^18^ The red marking shows the step in the reaction mechanism where the H^+^ ions are generated, which significantly influence the reaction pH value.

As a first step, a reference experiment was established to observe the behavior of pH and particle formation in water without any pH control. For this, aqueous solutions of l-dopa and tyrosinase were mixed under ambient conditions. Due to its acidic behavior in water, the pH of the l-dopa solution is between 5 and 6 with the actual value depending on the l-dopa concentration. In contrast, the enzyme does not affect the pH value. After combining the l-dopa solution with the enzyme solution to induce melanin formation, a rapid drop in pH during the first minutes is observed. This drop is due to the generation of protons during the spontaneous ring closure reaction from l-dopaquinone to leucodopachrome (Figure 2). Curiously, the fact of proton release is always found in the mechanisms but never attracted specific attention.

The reaction to dopachrome and thus also to its precursor leucodopachrome takes place very quickly,^18,38^ which can also be observed visually by the orange coloration of the reaction solution caused by dopachrome. This fact enables analysis of the reaction progress by means of UV/vis spectroscopy. In the following, transmission of the reaction at two fixed wavelengths was measured over time. The wavelength of 475 nm corresponds to the peak maximum of the dopachrome spectrum and was selected to observe the molecular buildup, and the wavelength of 633 nm was used to monitor the reaction of the supramolecular structure. The value 633 nm also corresponds to the wavelength of the laser used for light scattering data. Figure 2 shows the evolution of the pH value in direct comparison with the results of a light scattering measurement and the transmission at the two different wavelengths for the first 30 min of the reference experiment.

The dopachrome-based transmission at 475 nm runs almost parallel to the reaction pH during the observed time with the drop of both parameters being the strongest during the first 5 min. The strong initial decrease in the transmission at 475 nm is due to the very fast formation of the orange-colored dopachrome. The coloration of the samples from colorless to intense orange within 5 min shows that dopachrome is formed very quickly. The fact that the intense orange coloration still remains, which is supported by the constant value of the transmission, shows that even after the onset of particle formation, dopachrome continues. This also agrees with the findings of Ito et al.,^18,38^ which shows that the cyclization step from l-dopaquinone H^+^ to cyclodopa is the slowest step in the melanin buildup reaction. Molecular and supramolecular buildup takes place simultaneously in the observed time of 30 min.

A direct comparison of the reaction progress via UV/vis with the pH over time confirms that the sharp decrease in pH correlates directly with the formation of the precursors and not with the supramolecular buildup of melanin particles. In fact, the transmission at 633 nm only slightly changes during the first minutes. It sharply decreases from 100 to 95% transmission and then increases again up to a value of 97% and passes a shallow maximum at a reaction time of about 6 min. The loss of transmission at the beginning is on the one hand due to the mixing of the enzyme with the l-dopa solution but also caused by the absorption due to dopachrome. This second much slower decrease starting beyond 6 min is due to the formation of melanin.

The observation of particle growth by means of time-resolved light scattering in direct comparison with the pH value confirms this interpretation. The molar mass of eumelanin particles increases after a lag-phase of 2 min. The values of *R*_g_ are initially close to 50 nm, then increase up to 200 nm within less than 10 min, and finally reach a plateau. Even after *R*_g_ reaches a plateau, the particle mass increases even further although with a lower gradient. During the first minutes, simultaneous growth of particle size and mass signifies particle growth. The ongoing increase of *M*_w_ beyond 10 min (with *R*_g_ = constant) can be attributed to an increase of the overall number of particles, with sizes around 200 nm. As observed during minute 2 to minute 6, the formation of first individual particles takes roughly at 4 min, whereas the entire process goes on for hours. Beyond 6 min, growth of individual particles is overlaid by the addition of further particles. This behavior is analogue to our previously described investigation of the final type-B particles.^21^

### Influence of pH Control on the Particle Type

Although the usual pH shift in the melanin synthesis originates from the low molecular reaction cascade, it nevertheless seems to be an important impact factor on the resulting supramolecular structure of the melanin particles. As seen before, a final pH of around 4.0 results if no action to control the pH has been undertaken. In this case, type-B particles are gained, which is also the most common natural structure. Controlling and adapting the final pH of the reaction could therefore offer a feasible route to target stable substructures, i.e., type-A particles or even protoparticles.

To investigate this rationale, small amounts of sodium hydroxide solutions ([NaOH] = 0.1 M) were added to the reaction mixture. By this, the natural pH-drop is countered to reach a final value in the neutral range. The main idea was to address a pH close to the optimum of enzyme activity, which is 6.8. Addition of NaOH was made immediately after the initial rapid pH decrease at the beginning of the reaction cascade. In total, three different amounts (35, 40 and 45 μL) were added in two steps to avoid deactivation of the enzyme by an overreaching pH.

**Figure 3:**
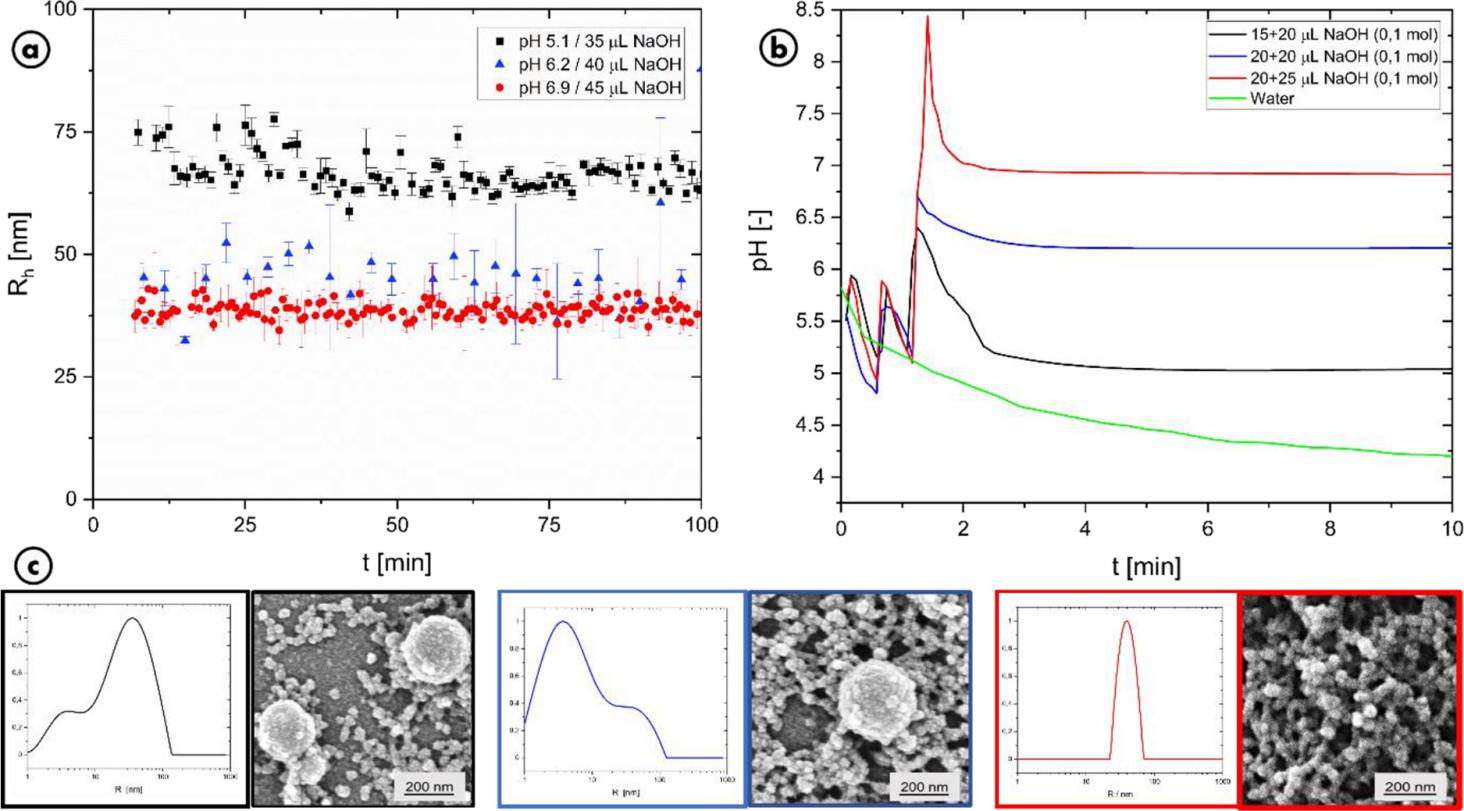
Influence of different amounts of NaOH being added immediately after mixing the l-dopa solution with the enzyme solution. a.) Evolution of the hydrodynamic radius over time from DLS with amounts of 35 μL (black square), 40 μL (blue triangle), and 45 μL (red circle) of 0.1 M NaOH and b.) the associated pH values over time. c.) In addition, the size distribution weighted by scattering intensity from CONTIN analysis measured at 90° is shown for each sample. Complementary SEM pictures as well as the results of the CONTIN analysis are marked by the colored frames of the pictures. The experiments were all carried out with 0.1 g/L l-dopa and 0.05 g/L tyrosinase.

As is demonstrated in Figure 3, this protocol indeed enables the targeted synthesis of stable type-A particle dispersions. The evolution of pH during all three experiments shows a strong back and forth behavior in the initial phase. However, after a few minutes, a stable pH (5.1, 6.2, and 6.9, respectively) is gained, which is always significantly higher than in the reference experiment. The fact that the pH plateau is reached after shorter reaction times compared to the reference experiment also indicates an overall expedited kinetics. This is plausible as the pH is closer to the optimum for enzyme activity. In the end, three new final pH values have been achieved. The consequences on the supramolecular buildup were subsequently investigated by a combination of time-resolved light scattering and electron microscopy (Figure 3).

Time-resolved measurement of the hydrodynamic radii for the three final pH values clearly demonstrates a high sensitivity of the system toward acidity. A clear decrease in mean particle size is seen. Without pH control, a mean size of around 200 nm was seen previously (Figure 2). With pH control, the average values are found to be 65, 45, and 30 nm, respectively. At first sight, this seems to contradict the previous findings, which clearly favor defined, narrow-sized particle substructures. However, a CONTIN analysis of the dynamic light scattering data reveals validity of the substructure model. Here, a clear bimodal distribution is seen for the lower pH values. From this, it is apparent that in fact, a mixture of type-A and type-B particles is produced in these experiments without any other intermediate-sized species. These results are further confirmed by the SEM pictures of the dried samples. Again, two different particle species are observed, which perfectly correspond to the type-A and type-B variants of eumelanin.^21,30^ Both analyses also show a comparatively large proportion of the type-B particles for the lowest pH and a decreased amount of type-B for the medium pH. At the highest pH (6.9) finally, no type-B particles are found anymore. This also explains the much less scattering of the time-resolved radii in the last experiment.

**Figure 4:**
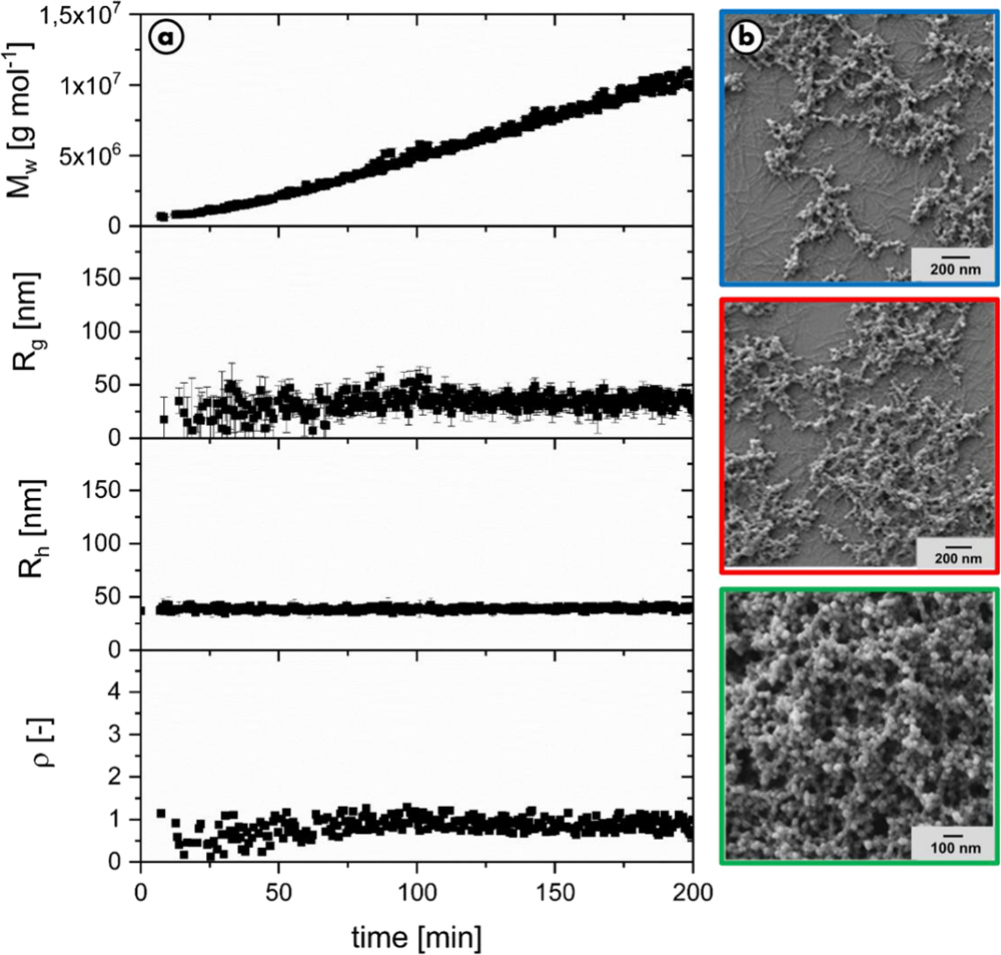
a.) Evolution of molar mass, radius of gyration, hydrodynamic radius, and structure-sensitive parameter ρ over time measured with time-resolved static and dynamic light scattering complemented with b.) SEM pictures showing snapshots at aggregation times indicated by the colored circles. The experiment was carried out with 0.1 g/L dopa, 0.05 g/L tyrosinase, and 45 μL of sodium hydroxide solution to raise the pH.

Based on these results, a refined protocol was established to enable a more reliable preparation of type-A dispersions in pure form. This protocol relies on a split addition of the hydroxide solution in two portions of 20 μL after 30 s and 25 μL after 1 min, resulting in a total addition of 45 μL. This experiment is again analyzed by the efficient and successful combination of time-resolved light scattering and SEM (Figure 4).

The molar mass of eumelanin particles increases at an accelerating growth rate during the first 25 min, after which a constant rate is adopted. Already, the very first values of *R*_g_ appearing at 25 min are close to 25 nm and remain at this constant value over the entire observed time of about 3 h. No formation of larger particles is observed. The behavior of *R*_h_ is in analogy to *R*_g_; however, the numerical values for R_h_ are slightly higher and around 35 nm. The further increase of *M*_w_ at constant radii is once again attributed to an increasing overall number of particles, which affects the weight average particle mass more significantly than the *z*-averaged square of the size values around 25 nm.

As the first observable radii have already adopted the final value of type-A particles, the formation of the individual type-A particle, supposedly by aggregation of protoparticles, is obviously so fast that it cannot be resolved with this approach. To support the light scattering data, the samples were again dried at various stages and pictured via SEM, which are also shown in Figure 4. In all images, solely type-A particles are seen. Only their number increases with the ongoing reaction time without any change of their size. In more detail, SEM showed a particle size of 30–40 nm in diameter, slightly smaller than determined by means of light scattering. The small difference is most likely due to the dried state of the particles in the microscopy images. To conclude, the entire results perfectly confirm our previous findings on this matter.^30^

Already at this point, it can be stated that stable dispersions of pure type-A eumelanin particles are accessible by the proper choice of experimental conditions, in particular the final pH of the reaction mixture. It was shown that addition of a well-defined amount of NaOH inhibits the supramolecular buildup at the level of type-A particles. However, pH adjustment by means of hydroxides was found to be tricky as the exact amount of NaOH must be fine-tuned with extreme sensitivity each time any changes to other reaction conditions are made, like for instance the amount of starting material. The inadequate pH at the beginning of the reactions is an important factor that should ideally be eliminated for a defined protocol.

### Control of the Reaction pH with MES Saline Buffer

Utilization of a suitable buffer is expected to accommodate the aforementioned issues and to bring more reliability and flexibility to the targeted type-A particle synthesis. Therefore, a suitable buffer system for melanin synthesis at the desired pH values had to be identified. The prerequisites are compatibility with all the reaction components, especially the enzyme, and the manifold reactions taking place. This is not entirely trivial as many biological buffer systems in the targeted pH range (4–8) are either not suitable due to the undesirable side reactions with aldehydes and ketones, incompatible with oxidation reactions, or react specifically with metal cations such as the copper complex present in the tyrosinase. For example, phosphate buffers with and without sodium chloride were investigated and later excluded as they induced aggregation of the tyrosinase in the respective pH range. As the next promising candidate known for its compatibility with almost all biological systems, saline MES buffers (2-(*N*-morpholino) ethane sulfonic acid) were investigated. MES was first tested for its compatibility with the enzyme and l-dopa. For this purpose, it was checked whether both components are completely dissolved in the aqueous buffer systems without aggregating, denaturing, or precipitating. A 24 h study showed no difference in solubility of the enzyme and l-dopa in both water and MES buffer. The MES buffer was thus attested for compatibility with the melanin synthesis.

**Figure 5:**
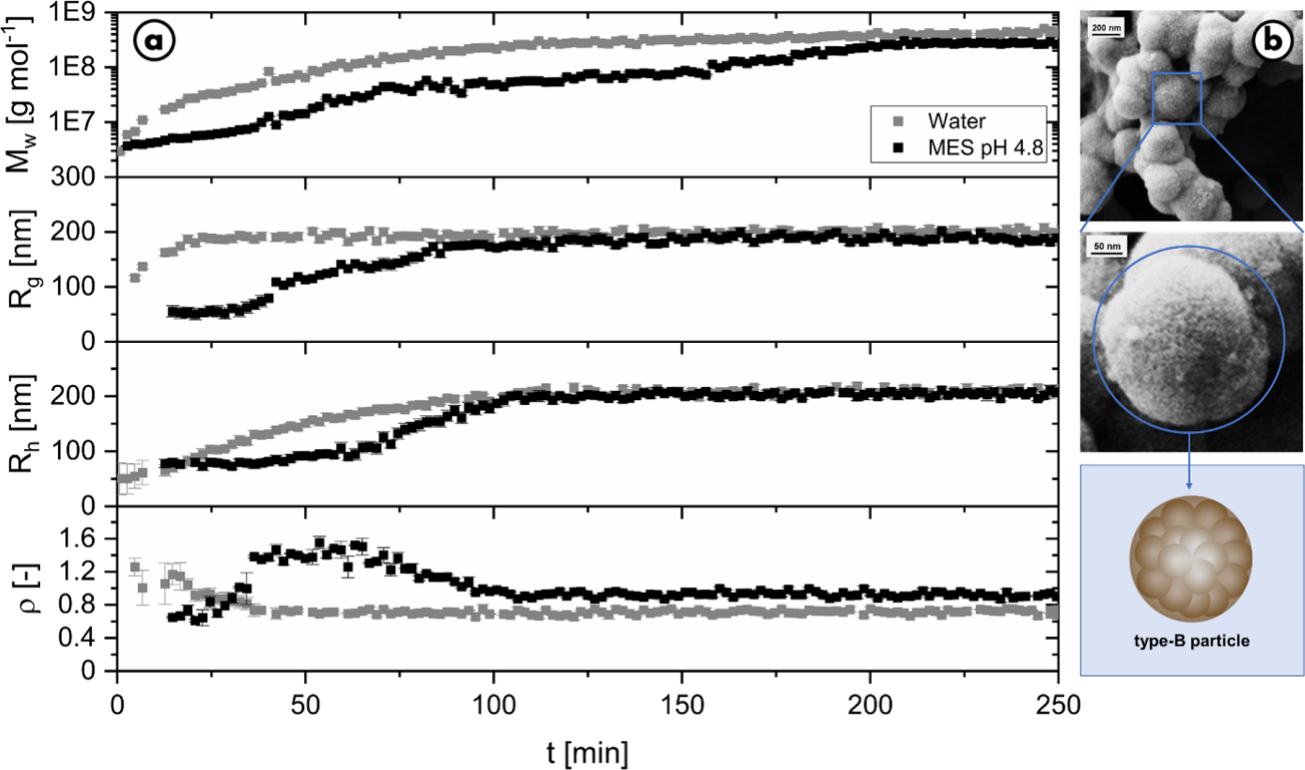
a) Evolution of molar mass, radius of gyration, hydrodynamic radius, and structure-sensitive parameter ρ over time for the standard reaction in water, and in MES buffer with a pH of 4.8, close to the pH during the standard reaction, with all data measured with time-resolved static and dynamic light scattering. b) The resulting SEM pictures showing the final type-B particles. The experiments were carried out with 0.1 g/L l-dopa and 0.05 g/L tyrosinase in water and in 30 mM MES.

To further verify the applicability of MES, type-B particles were again prepared in a similar way as in the previous reference experiment but in the MES buffer instead of water. To get as close as possible to the conditions of the reference experiment in water (final pH = 4.0), the lowest possible pH of MES (4.8) was applied. Figure 5 shows a direct comparison of the reaction in water with the reaction in MES buffer at pH 4.8.

It is observed that the synthesis of type-B particles was successful using MES buffer at a pH of 4.8. MES buffer only caused minor changes in the reaction. A direct comparison with the reference experiment in Figure 5 shows that the particle formation in the MES buffer system is slowed down significantly. The molar mass *M*_w_ grows steadily from the start of the reaction but at a significantly slower rate than in the reference reaction. The retardation observed in the presence of MES buffer even made accessible the radii of the type-A particles as initial intermediates, not observed in the reference reaction. The structure-sensitive ratio ρ complements these findings. In MES buffer, ρ starts with a value of 0.7 indicating compact spheres and then increases to a value of 1.3. The appearance of a maximum at ρ = 1.3 can be attributed to fluffy intermediate aggregates of the type-A particles, which have not yet reached the final size of the type-B particles. As the amount of the completed, spherically shaped type-B particles increases, the lower limit of ρ < 1 is re-approached. The reference reaction in water is much faster, and the ρ-value starts directly with a value of 1.3, which now steadily decreases until a constant value of 0.7 is reached suggesting that beyond 50 min, the data is dominated by the ongoing completion of compact type-B particles at the expense of type-A particles. Successful preparation of type-B particles in MES buffer is also reflected in the SEM images as the electron microscopic images of the MES samples show the same 200 nm type-B particles as in the water measurement (Figure 5).

To conclude, it could be shown that the addition of MES buffer to the recipe of the reference reaction (in water) significantly slows down the reaction. In MES, even the type-A particles, which are initially formed, were detected as an intermediate stage prior to aggregating toward the final type-B particles. The slowdown in particle synthesis could be due to the higher pH and stabilization of the intermediates such as dopachrome or the actual monomers DHI and DHICA. In the MES system, the pH could only be buffered at the lowest possible limit of 4.8, whereas the pH of the reference experiment has decreased to about pH 4.0 after a reaction time of 5 min and remains there for the rest of the observed time.

### Targeted Type-A Particle Synthesis in MES Buffer

**Figure 6:**
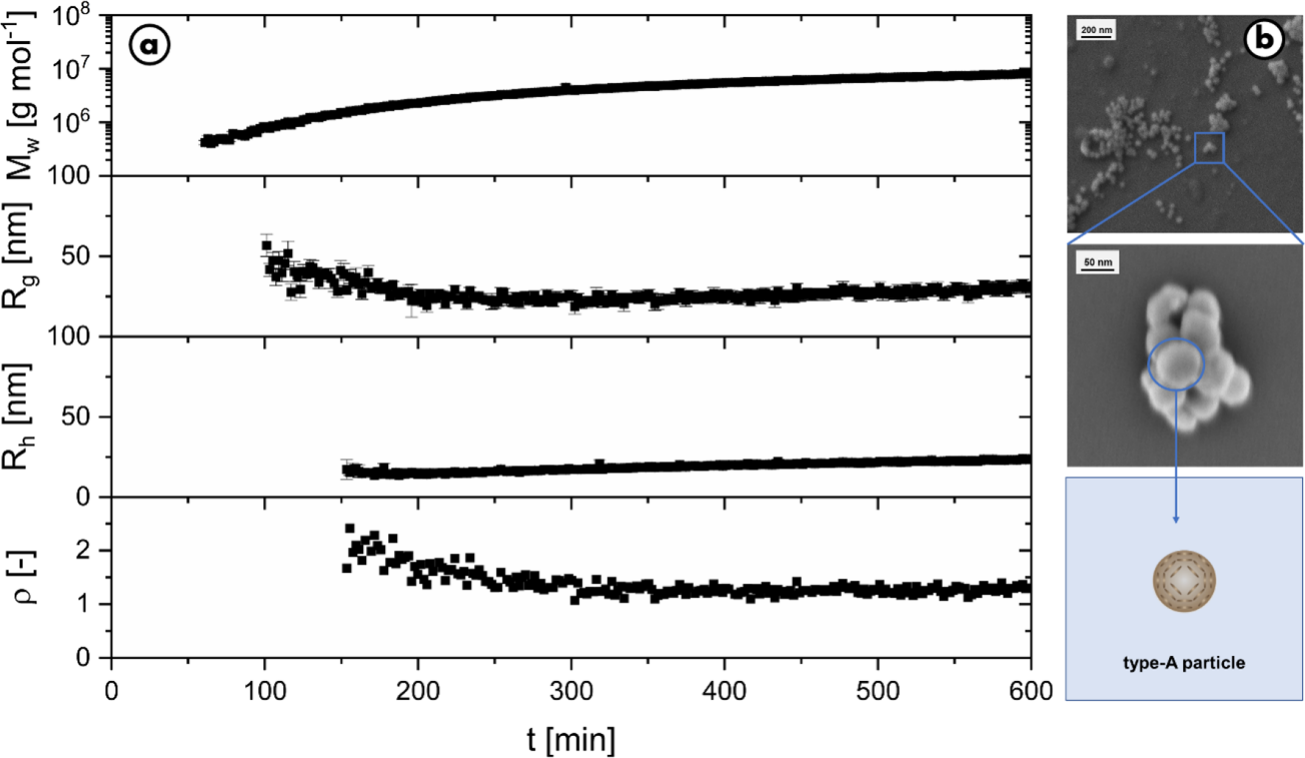
Evolution of molar mass, radius of gyration, hydrodynamic radius, and structure-sensitive parameter ρ over time for the reaction in MES buffer at a pH of 6.2, measured with time-resolved static and dynamic light scattering, targeting with the corresponding final type-A particle shown by SEM. The experiments were carried out with 0.1 g/L l-dopa and 0.5 g/L tyrosinase in 15 mM MES.

As the general compatibility of MES buffer in enzyme-mediated melanin synthesis is now confirmed, a protocol for the main goal, the targeted synthesis of type-A particles is established next. The previous experiments revealed that the pH for the targeted synthesis of type-A particles must be above 6.0. The new experiment was therefore conducted with a 15 mM MES buffer at pH 6.2. All other parameters remain unchanged (l-dopa = 0.1 g/L and enzyme = 0.05 g/L). Figure 6 shows an increase of molar mass after a lag phase of nearly 50 min. After a lag phase of nearly 150 min, the first values for both *R*_g_ and *R*_h_ are observed close to 25 nm. Both radii remain at this value for the rest of the observed time. After the radii reach their plateau values, the particle mass keeps on growing, although with a decreasing gradient, until it also reaches a constant value after about 300 min. This ongoing increase of *M*_w_ beyond 150 min (with *R*_g_ and *R*_h_ = constant) can again be attributed to the increase of the overall number of particles, all adopting the final radii close to 25 nm. The values determined for the structure-sensitive parameter ρ amount to a constant value of 1.2, which does not correspond to the expected value for compact spheres of 0.7. However in contrast, the SEM images confirm once again the well-known shape of compact spheres with a uniform size of no more than 50 nm in diameter. This discrepancy might be explained by the fact that the particles in solution are swollen, in contrast to the dried particles for the SEM measurements. Also, the reaction is much slower than before; it is therefore possible that a previously hidden consolidation becomes observable here. Such a behavior would be similar to the previously seen consolidation in the step from type-A to type-B.^21^

In summary, targeted type-A synthesis is possible in an MES-buffered system at pH 6.2. This protocol offers an overall enhanced control with less delicate experimental handling and still reliably inhibits the next aggregation step. The process is easily applied and allows access to the previously unknown stable dispersions of the isolated eumelanin type-A substructures.

### Reactivation of the Aggregation Step toward Type-B Particles

Now that it has been shown that a defined reaction pH can be utilized to inhibit the final supramolecular step in the eumelanin synthesis, the next logical question is whether this inhibition is reversible or irreversible.

To address this question, the stable type-A particles in MES buffer at pH 6.2 were first stored for 24 h. Next, a possible reactivation is triggered by lowering the pH value from 6.2 to 4.8 using 0.1 M HCl. Special care must be given to the proper amount of 0.1 M HCl as too low pH values would lead to precipitation of melanin.

**Figure 7:**
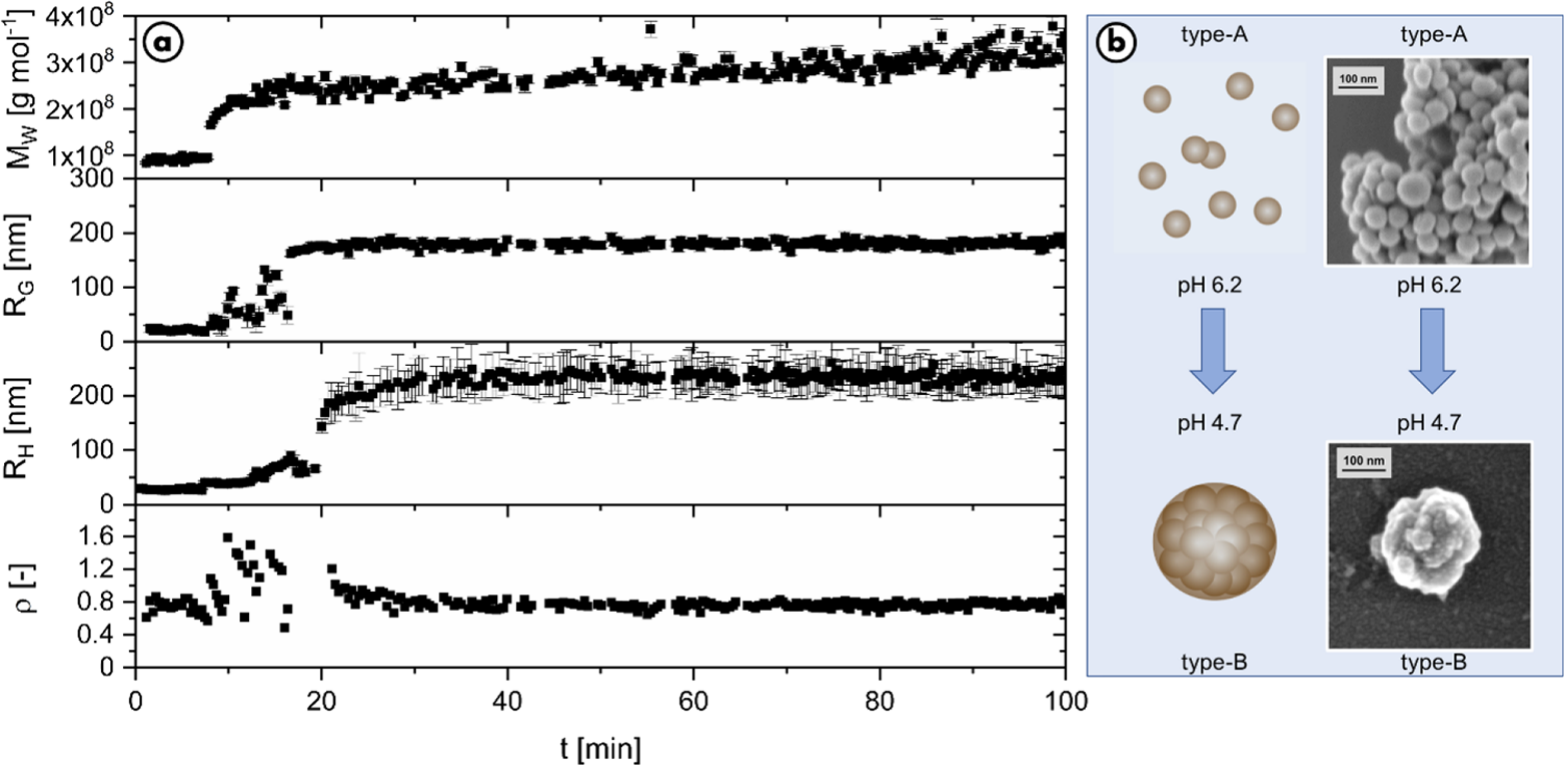
Initiation of type-B particle formation via a pH drop from 6.2 to 4.8 in a solution of type-A particles prepared via the MES recipe and aged for 24 h. a.) Evolution of molar mass *M*_w_, radius of gyration *R*_g_, hydrodynamic radius *R*_h_, and structure-sensitive parameter ρ over time prior to and after adding 31 μL of HCl (8 min) to trigger particle growth. b.) The schematic representation shows the reshaping of the particles due to the change in pH.

The results are presented in Figure 7. First, the dispersion aged for 24 h was characterized for 7 min prior to adjusting the pH to 4.8. Recovery of the mass values and radii measured confirms stability of the type-A particle dispersion for at least 24 h. Curiously, the value for the structure-sensitive factor ρ is reduced to 0.7 after aging. This is another hint for a possible consolidation behavior as described before and now also supports the spherical shape seen in the SEM pictures. After the predetermined pH shift from 6.2 to 4.8 at 8 min, the system reacts immediately. The evolution of the particle mass and the radii is close to the trends shown in Figure 4b and indeed leads to type-B particles. This clearly demonstrates that the inhibition of type-A particle aggregation is reversible and clearly dependent on the pH at any current time.

## Conclusions

It has been shown that the supramolecular buildup of enzyme-mediated eumelanin can, at least partially, be controlled by application of defined pH levels. As a first novel insight, it was shown that the release of protons during the low molecular reaction cascade significantly lowers the pH. This effect seems to be important for the formation of the natural type-B species as they are only formed at pH values around 4.

A deliberate increase of the pH reduces (or significantly retards) the type-B aggregation, and the isolated type-A particles become observable. Above a pH of 6.0, solely type-A particles are observed even after 24 h of aging. The latter strongly implies complete inhibition of the final aggregation step. The experimental procedure can be made more convenient by application of a saline MES buffer for pH control. It has furthermore been demonstrated that the inhibition is reversible even after 24 h. If the pH is again lowered to 4.0, aggregation toward the type-B particles immediately recommences.

These findings define the first protocol to synthesize stable dispersions of type-A eumelanin and also demonstrate the importance of the pH in the buildup of melanin particles. Further research will show if a similar behavior can be identified for the other aggregation steps in the supramolecular buildup. This opens up the potential to gain protoparticles or even non-particular oligomers toward various high-tech applications and a profound understanding of the evasive supramolecular buildup of eumelanin. Future research could also investigate if lowering of the pH is likewise achieved within the biological melanosome by the molecular reaction cascade and which consequences might arise from this.
